# Unraveling the Geometry of Visual Relational Reasoning

**Published:** 2025-02-24

**Authors:** Jiaqi Shang, Gabriel Kreiman, Haim Sompolinsky

**Affiliations:** 1 Program in Neuroscience, Harvard Medical School, Boston, Massachusetts & 02115, United States.; 2 Boston Children’s Hospital, Harvard Medical School, Boston, Massachusetts & 02115, United States.; 3 Center for Brains, Minds, and Machines, Cambridge, Massachusetts & 02139, United States.; 4 Center for Brain Science, Harvard University, Cambridge, Massachusetts & 02138, United States.; 5 Edmond and Lily Safra Center for Brain Sciences, Hebrew University, Jerusalem & 9190401, Israel.

## Abstract

Humans and other animals readily generalize abstract relations, such as recognizing *constant* in shape or color, whereas neural networks struggle. To investigate how neural networks generalize abstract relations, we introduce *SimplifiedRPM*, a novel benchmark for systematic evaluation. In parallel, we conduct human experiments to benchmark relational difficulty, enabling direct model-human comparisons. Testing four architectures—ResNet-50, Vision Transformer, Wild Relation Network, and Scattering Compositional Learner (SCL)—we find that SCL best aligns with human behavior and generalizes best. Building on a geometric theory of neural representations, we show representational geometries that predict generalization. Layer-wise analysis reveals distinct relational reasoning strategies across models and suggests a trade-off where unseen rule representations compress into training-shaped subspaces. Guided by our geometric perspective, we propose and evaluate SNRloss, a novel objective balancing representation geometry. Our findings offer geometric insights into how neural networks generalize abstract relations, paving the way for more human-like visual reasoning in AI.

## Introduction

Humans and other animals demonstrate a remarkable ability to identify and generalize abstract relations across diverse contexts. For example, recognizing the relation “constant” allows individuals to flexibly apply it to different attributes, such as a row of objects sharing the same color or shape. Furthermore, one can readily apply the same abstract relation to novel attributes never encountered before. This ability to generalize abstract relations underpins flexible reasoning, supports complex problem-solving, and enables intelligent behavior in novel situations.

A central challenge in cognitive neuroscience and artificial intelligence is to develop neural network models that can achieve similar abstract relation generalization. The Raven’s Progressive Matrices (RPM) task is a widely used benchmark for assessing abstract relational reasoning ability (1). In this task, participants view a 3×3 grid of images with the bottom-right panel missing. The goal is to identify the relational rules that govern the grid and select the correct missing panel based on these inferred rules. Inspired by this task, researchers have developed large-scale RPM-style datasets, such as Procedurally Generated Matrices (PGM) (2) and the Relational and Analogical Visual rEasoNing (RAVEN) dataset (3,4), and have trained various neural architectures to solve them (2–14). While many of these models achieve high test accuracy on the relational rules they are trained on, they struggle to generalize to unseen rules (15). The absence of human behavioral data on the same generalization tasks measured in neural networks further complicates the interpretation of existing results.

This limitation raises a fundamental question: What mechanisms enable or constrain abstract relation generalization in neural networks? While prior studies have documented performance levels, the representational factors shaping generalization remain poorly understood. In particular, it is unclear how learned representations encode relational structure, how these representations differ across neural network architectures, and what geometric constraints govern generalization.

In this work, we introduce a geometric framework to systematically analyze the structure of relational representations in neural networks. To compare quantitatively network generalization performance against humans, we introduce a new benchmark, the Simplified Raven’s Progressive Matrices (*SimplifiedRPM*) task. Using this benchmark, we compare four representative neural networks spanning different reasoning strategies. ResNet-50 (16), Vision Transformer (17), Wild Relation Network (2), and Scattering Compositional Learner (SCL) (5). We conduct human experiments, concluding that SCL aligns most closely with human relational reasoning.

*SimplifiedRPM* enables applying a geometric theory (18) originally developed for generalization in visual object classification tasks to the reasoning domain. Specifically, we propose that the reasoning performance of neural networks can be attributed to the geometry of their emergent “rule manifolds”. We demonstrate that this geometric theory precisely predicts empirical generalization error in neural networks, establishing a direct link between the geometry of rule manifolds for unseen relations and generalization performance. Our analysis highlights two key geometric factors, signal and dimensionality, which interact to determine the generalizability of a network. Importantly, we observe a trade-off between signal and dimensionality, driven by a structured compression mechanism, where networks amplify the signal by constraining all class manifolds—including unseen ones—into a low-dimensional subspace spanned by the principal components of the training rules. Motivated by this theory, we introduce *SNRloss*, a new network training objective explicitly designed to optimize the representation geometry and show that this loss function leads to adequate generalization performance with balanced geometric features. Overall, this work provides new insights into the mechanisms underlying relational reasoning through the lens of representation geometry.

### Evaluating Deep Neural Networks in Relational Reasoning

**Figure 1:**
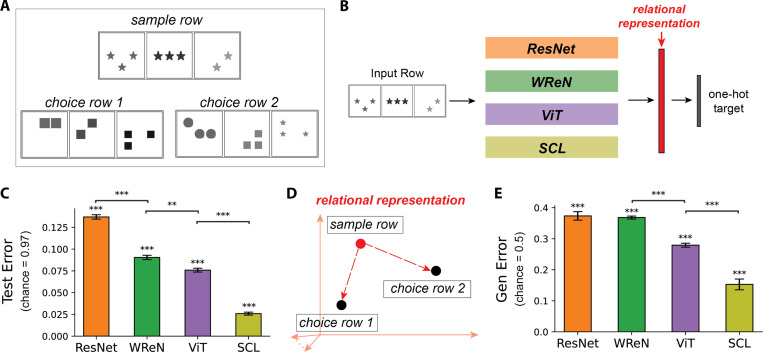
The *SimplifiedRPM* dataset helps evaluate neural networks in relational reasoning and generalization. (**A**) Example *SimplifiedRPM* trial with “constant shape“ rule. Choice 1 is correct; Choice 2 follows “constant number” and is incorrect. (**B**) We evaluate four representative models on the *SimplifiedRPM* task: ResNet-50 (orange) (16), WReN (green) (2), ViT (purple) (17), and SCL (yellow-green) (5). Each model encodes relational rules in rows using high-dimensional relational representations (red) and is trained end-to-end. A linear classifier is appended during training to categorize relational rules. See Methods for details. (**C**) Test errors for four models across 15 random splits of training and held-out rules, with error bars denoting s.e.m. All models perform significantly above chance (0.97) based on a one-sample t-test: ResNet (*t*(14) = −312.77, *p* = 1.2×10^−28^),WReN(*t*(14) = −343.78, *p* = 3.4×10^−29^),ViT(*t*(14) = −388.11, *p* = 6.2×10^−30^) and SCL (*t*(14) = −542.53, *p* = 5.7 × 10^−32^). Pairwise Wilcoxon tests confirm SCL achieves the lowest test error, followed by ViT, WReN, and ResNet (p-values: SCL vs. ViT, 3.0 × 10^−5^; ViT vs. WReN, 1.6×10^−3^; WReN vs. ResNet, 3.0×10^−5^). (**D**) Schematic illustrating distances (red arrows) between sample and choice row relational representations. The trial is solved correctly if the correct choice row representation is closer to the sample row than the incorrect choice. (**E**) Generalization error of four models on *SimplifiedRPM*, averaged over 15 random training and held-out rule splits. Error bars denote s.e.m. All models perform significantly above chance (0.5) based on a one-sample t-test: ResNet (*t*(14) = −8.93, *p* = 1.8 × 10^−7^), WReN (*t*(14) = −26.39, *p* = 1.2 × 10^−13^), ViT (*t*(14) = −33.45, *p* = 4.6×10^−15^), and SCL (*t*(14) = −19.35, *p* = 8.3×10^−12^). Pairwise Wilcoxon tests confirm SCL achieves the lowest error, followed by ViT, while WReN and ResNet show similarly high errors (p-values: SCL vs. ViT, 6.1 × 10^−5^; ViT vs. WReN, 3.0 × 10^−5^). Asterisks indicate statistical significance: ** (*p* < 0.01), *** (*p* < 0.001).

To systematically assess neural networks’ ability to generalize abstract relations, we developed the Simplified Raven’s Progressive Matrices (*SimplifiedRPM*) task (Fig. 1A). Inspired by the original RPM task (1), *SimplifiedRPM* requires models to infer a relational rule from a *sample row* (Fig. 1A, top) and determine which of two *choice rows* (Fig. 1A, bottom) follows the same rule. Each row consists of three *panels* with abstract objects, such as triangles or circles, arranged according to a relational rule. For example, the sample row in Fig. 1A illustrates the “constant shape” rule, where all objects share the same shape while other attributes, such as number or position, vary. *SimplifiedRPM* refines the original RPM task (1) by reducing answer choices from eight to two, mitigating selection bias exploitation (4), and presenting a single example row instead of two to remove multi-row comparisons. These changes maintain RPM’s core challenge while enabling a more focused assessment of relational reasoning.

We construct a dataset for evaluating abstract relation generalization in the *SimplifiedRPM* task, comprising rows governed by 40 distinct relational rules. Each rule applies an abstract relation (e.g., constant) to an object attribute (e.g., shape). The abstract relations and attributes are selected to be consistent with those used in human experiments (1,19) while maximizing the number of rules for comprehensive evaluation. Importantly, each row corresponds to a unique rule. We generate many rows per rule, resulting in a dataset of 400k training rows, 40k validation rows, and 40k test rows. To construct a *SimplifiedRPM* task trial, a sample row is selected that follows one rule. A correct choice is drawn from the same rule, while an incorrect choice is drawn from a different rule. Further details are provided in the Methods section.

We assess models’ ability to generalize abstract relations using a held-out rule split, where 5 out of 40 relational rules are excluded from training. During training, models learn relational structures from the remaining 35 rules. For instance, a model trained on ”constant number” and ”OR shape” may never encounter ”constant shape.” After training, their performance is evaluated on the five withheld rules using the *SimplifiedRPM* task. This design addresses limitations in the existing RPM benchmarks. RAVEN (3) lacks a split where some test rules are entirely unseen during training. Although PGM’s Held-out Triplet split withholds certain rules, test rows still combine seen and unseen rules, allowing models to rely on familiar rules. In contrast, *SimplifiedRPM* enforces a strict separation between training and test rules, ensuring a more rigorous evaluation of relational generalization. By systematically varying which rules are withheld, our benchmark provides a comprehensive assessment of models’ ability to generalize across different types of abstract relations.

We evaluate four representative models on the *SimplifiedRPM* task: ResNet-50 (16) and ViT (17), two benchmark vision models, and WReN (2) and SCL (5), which are explicitly designed for relational reasoning. These models employ different computational strategies: ResNet-50 applies a series of convolutional layers across all panels simultaneously, WReN computes pairwise panel relationships and aggregates them to infer underlying rules, ViT leverages self-attention to capture relational dependencies, and SCL employs a shared relational module to improve abstract relation recognition across object attributes. Together, these models represent diverse approaches to solving RPM tasks. All models are randomly initialized and trained end-to-end. Further details on the model architecture and hyperparameters are provided in the Methods section.

The models process a row to generate a high-dimensional *relational representation* of its underlying relational rule (Fig. 1B). To train the models on 35 relational rules, we appended a linear classification layer to the relational representations and optimized them end-to-end using cross-entropy loss with one-hot rule labels. Fig. 1C shows test errors across 15 random training and held-out rules splits. All models successfully learned the relational rules, achieving test errors well below the chance level of 34/35 = 0.97. Among them, SCL achieved the lowest test error, followed by ViT, WReN, and ResNet-50.

We assess the trained models’ ability to generalize abstract relations to unseen rules using the *SimplifiedRPM* task. For each pair in the five held-out rules, we generate 500 trials by randomly selecting a sample row that follows one rule, pairing it with a correct choice from the same rule and an incorrect choice from a different rule. In each trial, the model is sequentially presented with the sample row, followed by the two choice rows. The model computes the Euclidean distance between the relational representation of the sample row and each choice row. A trial is deemed correct if the correct choice row is closer to the sample row than the incorrect one (Fig. 1D). All models outperformed the chance generalization error of 0.5 (Fig. 1E). Among them, SCL achieved the lowest generalization error, followed by ViT, WReN, and ResNet. Notably, SCL also outperformed the other models on the PGM benchmark’s held-out rule split (15), aligning with our observed generalization order.

### SCL Captures Human-like Differential Relational Rule Difficulties

**Figure 2:**
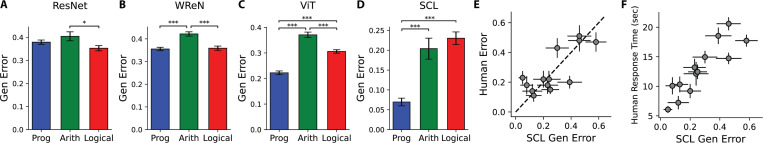
SCL Captures Human-like Differential Relational Rule Difficulties. (**A-D**) Generalization errors of four models on the *SimplifiedRPM* task, evaluated across three abstract relation types: Progression (blue), Arithmetic (green), and Logical (red). The chance level is 0.5. Each bar represents the average error for trials where the correct rule of the sample row fell into one of these relation types, with error bars denoting s.e.m. (**A**) ResNet exhibited significantly higher errors for arithmetic rules than logical rules (p = 0.019, Pairwise Wilcoxon test). (**B**) WReN showed significantly higher errors for arithmetic rules than both progression (*p* = 4.2 × 10^−6^) and logical (*p* = 9.1 × 10^−5^) rules. (**C**) ViT exhibited higher errors for arithmetic rules compared to both progression (*p* = 3.8 × 10^−17^) and logical (*p* = 7.7 × 10^−7^) rules. Additionally, logical rules had significantly higher errors than progression (*p* = 2.0 × 10^−12^). (**D**) SCL progression was lower than arithmetic (*p* = 2.1×10^−17^) and logical (*p* = 3.2×10^−8^). Statistical significance was assessed using Pairwise Wilcoxon tests. Asterisks indicate statistical significance: * (*p* < 0.01), *** (*p* < 0.001). (**E**) Comparison of the SCL model’s generalization errors against human performance on the *SimplifiedRPM* task across 13 selected rule pairs (chance level = 0.5). Each dot represents the average model (x-axis) or human (y-axis) error over 10 questions for a given rule pair, with error bars indicating the s.e.m. The dashed line represents the line of identity. (**F**) Correlation between the SCL model’s generalization error (x-axis) and human response times (y-axis) for the 13 selected rule pairs. Each dot represents the average model error and corresponding human response time for a given rule pair, with error bars indicating the s.e.m.

Analyzing only the averaged generalization error is insufficient to fully assess neural networks’ ability to generalize abstract relations, as a model may perform well overall but struggle with rules of specific relation types. To gain deeper insight, we analyzed generalization errors separately for trials where the correct rule of the sample row fell into one of three abstract relation types: progression, arithmetic, or logical. We observed distinct error patterns across models (Fig. 2A–D). ResNet had higher errors for arithmetic than logical rules (Fig. 2A). WReN showed the highest errors for arithmetic, performing worse than progression and logical rules (Fig. 2B). ViT struggled most with arithmetic, while logical rules had higher errors than progression (Fig. 2C). In contrast, SCL showed similarly high errors for arithmetic and logical rules but significantly lower errors for progression (Fig. 2D).

Interestingly, while arithmetic rules posed the greatest challenge for models to generalize, this difficulty did not arise from poor learning of the rules. Instead, it highlights the model’s inability to generalize the arithmetic relation to new attributes. When evaluating test errors—the classification error on novel rows following the trained rules—arithmetic rules consistently had much lower errors than logical rules, which exhibited the highest errors across models (figure S1).

Human performance on the original RPM task (1) follows a hierarchy of difficulty, with progression rules the easiest and logical rules the hardest (19–22). Unlike models, humans do not receive explicit training on these rules before taking the task (23,24). To evaluate whether models exhibit human-like reasoning, we compare their ability to generalize to novel rules against human performance.-Among the models examined, only the SCL model replicates the human difficulty pattern across relation types (Fig. 2D). To more rigorously test this alignment, we conducted additional human studies using the *SimplifiedRPM* task, which allows a direct comparison of abstract relation generalization between humans and models.

To encompass a range of task difficulties, we sampled 130 trials of the *SimplifiedRPM* task, spanning 13 relational rule pairs with 10 questions per pair. We recruited *n* = 25 MTurk participants, each of whom answered 60 randomly selected questions from this pool to minimize sequential learning effects. Participants had unlimited time to respond and were compensated based on accuracy. For each trial, we recorded both accuracy and response time (see Methods for details). Human participants exhibited a mean task error of 0.270 ± 0.038 (mean±s.e.m.), significantly lower than the chance level of 0.5 (one-sample t-test, *t*(12) = −5.70, *p* = 4.9×10^−5^). The detailed 13 rule pairs included in the human experiment and their corresponding human performance are in table S2.

We evaluated the generalization errors of the SCL model on the same *SimplifiedRPM* trials used in human experiments. Specifically, we trained ten SCL models for each of the thirteen rule pairs, each using a different split of the forty rules in the dataset while ensuring that the target rule pair was always among the five held-out rules. We then measured the generalization error on the held-out pair using the same trials as those used in the human evaluation. The model achieved an average task error of 0.268 ± 0.043 (mean±s.e.m.), close to the human performance. Across the thirteen rule pairs, SCL models exhibited error patterns that correlated with those observed in human participants (Spearman’s rank correlation, *ρ* = 0.62, *p* = 0.02) (Fig. 2E).

Besides task errors, human response times—the total duration participants spent on each trial—were also strongly correlated with SCL generalization error across the thirteen rule pairs (Spearman’s rank correlation, *ρ* = 0.90, *p* = 1.8 × 10^−5^) (Fig. 2F). This finding suggests that participants allocated more time to solving trials that the model found more challenging. In summary, although trained solely to classify relational rules without explicit human-like guidance, the SCL model successfully captures the human order of difficulty. In contrast, other models deviate from human performance patterns (figure S2). This alignment of SCL with human performance motivates further exploration of the mechanisms underlying how neural networks perform relational reasoning.

### Geometric Theory of Rule Manifolds Accurately Predicts Error in the *SimplifiedRPM* Task

To investigate how neural networks achieve relational reasoning and generalize their learned representations to unseen relational rules, we leveraged a geometrical theory of few-shot learning (18). This theory provides a framework for linking errors in the Simplified RPM task to interpretable geometric properties of the relational representations.

**Figure 3:**
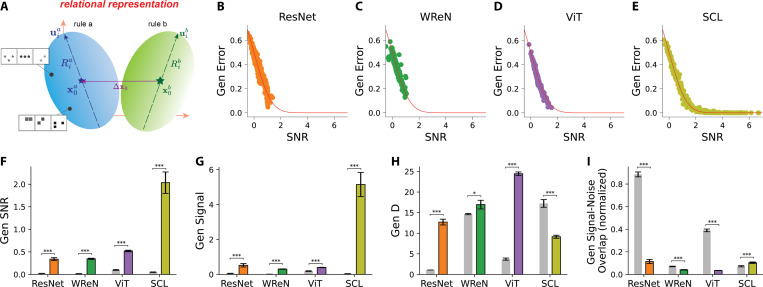
Geometric Theory of Rule Manifolds Accurately Predicts Error in the *SimplifiedRPM* Tasks. (**A**) Illustration of rule manifolds in the relational representation space. Orange axes denote representation dimensions. Each rule manifold consists of relational representations for rows that follow a specific rule. Two rule manifolds are shown: blue (rule a) and green (rule b) ellipsoids. The two black dots in rule a manifold represent example rows following rule A (e.g., constant shape). Each manifold is characterized by a centroid (${\text{x}}_{0}^{a}$ for rule a, blue star; ${\text{x}}_{0}^{b}$ for rule b, green star), representing the mean position of all relational representations following that rule. Variation is characterized by principal axes (${\text{u}}_{i}^{a}$ for the blue rule manifold a and ${\text{u}}_{i}^{b}$ for the green manifold b), shown as blue and green dashed arrows. Here, i=1,..,N, where N is the relational representation dimension. The extent of variation along each axis is quantified by the corresponding radii $R_{i}^{a}$ and $R_{i}^{b}$. Purple line represents signal $\text{Δ}{\text{x}}_{0}$. (**B-E**) Comparison of theoretical predictions (red lines) and empirical generalization errors (dots) across four models. Each dot represents a held-out rule pair, where the theoretical SNR predictions are computed using geometric measures (Equation 1), and the empirical generalization errors are averaged over 500 *SimplifiedRPM* trials. Each plot includes rule pairs from 15 distinct held-out rule splits. Each split consists of five held-out rules, resulting in 20 rule pairs per split. (**F-I**) Generalization SNR, signal, D, and signal-noise overlap (normalized by signal magnitude) for model relational representations on the held-out rules. The gray bars represent the untrained models’ representation at initialization, while the colored bars correspond to different models: orange for ResNet, green for WReN, purple for ViT, and yellow-green for SCL. Each bar shows the mean value averaged across 15 distinct held-out rule splits, with error bars indicating the s.e.m. Pairwise Wilcoxon tests assess whether the trained model terms differ from the untrained models. Asterisks indicate statistical significance: * (*p* < 0.01), *** (*p* < 0.001).

We define *rule manifolds* as the set of relational representations for rows that follow a specific rule (Fig. 3A). For a rule pair (a, b), we consider the average generalization error $\varepsilon_{a}$ on the *SimplifiedRPM* task across all trials where the sample and correct choice rows follow rule a, while the incorrect choice row follows rule b. Geometrically, $\varepsilon_{a}$ corresponds to the probability that a correct choice sampled from the rule a manifold is closer to an incorrect choice from the rule b manifold than to another correct choice from the rule a manifold.

Each rule manifold is defined by its centroid ${\text{x}}_{0}$ (depicted as blue and green stars in Fig. 3A), which represents the mean position of all representations that follow a given rule. The spread of representations within a manifold is captured by a set of principal axes ${\text{u}}_{i}$ (illustrated as blue and green dashed arrows in Fig. 3A), along which the variation is quantified by corresponding radii $R_{i}$. Here, i=1,…,N, where N denotes the total dimensionality of the relational representation space. The overall magnitude of this variation is measured by the mean squared radius given by: $R^{2}\equiv \frac{1}{N}\sum_{i=1}^{N}R_{i}^{2}$.

Although the rule manifolds may have complex shapes, the geometrical theory predicts that generalization error can be accurately estimated using only a few key geometric properties. Specifically, the generalization error, $\varepsilon_{a}$, for a given rule pair (a, b) is governed by the signal-to-noise ratio ${\text{SNR}}_{a}$, following: $\varepsilon_{a}=H\left({\text{SNR}}_{a}\right)$. Here, H(⋅) is the Gaussian tail function defined as $H(x)=\int_{x}^{\infty }dte^{-t^{2}/2}/\sqrt{2\pi }$. Higher ${\text{SNR}}_{a}$ values correspond to lower generalization errors. The dominant terms of ${\text{SNR}}_{a}$ are given by:

(1) $${\text{SNR}}_{a}=\frac{{\left\|\text{Δ}{\text{x}}_{0}\right\|}^{2}+R_{b}^{2}R_{a}^{2}-1}{\sqrt{D_{a}^{-1}+{\left\|\text{Δ}{\text{x}}_{0}\cdot {\text{U}}_{a}\right\|}^{2}+{\left\|\text{Δ}{\text{x}}_{0}\cdot {\text{U}}_{a}\right\|}^{2}}}$$

The ${\text{SNR}}_{a}$ depends on four key interpretable geometric terms. The first is the *signal*: ${\left\|\text{Δ}{\text{x}}_{0}\right\|}^{2}={\left\|{\text{x}}_{0}^{a}-{\text{x}}_{0}^{b}\right\|}^{2}/R_{a}^{2}$, which measures the squared Euclidean distance between the centroids (${\text{x}}_{0}^{a}$, ${\text{x}}_{0}^{b}$) of the two rule manifolds (depicted as purple line in Fig. 3A), normalized by the mean squared radius $R_{a}^{2}$ of rule a. A larger signal indicates greater separation between the rule a and rule b manifold centroids, leading to higher ${\text{SNR}}_{a}$ and lower generalization error. We denote $\text{Δ}{\text{x}}_{0}$ as the signal direction. The second term is the *bias*, $R_{b}^{2}R_{a}^{-2}-1$, which captures the relative difference in the sizes of two rule manifolds. When rule manifold a is larger than manifold b, the bias term is negative, predicting a lower ${\text{SNR}}_{a}$ for rule a. The third term, $D_{a}^{-1}$, represents the inverse of the dimensionality $D_{a}$ of the rule a manifold. $D_{a}$, known as the ”Participation ratio” (25), is defined as $D_{a}\equiv {\left(R_{a}^{2}\right)}^{2}/\sum_{i=1}^{N}{\left(R_{i}^{a}\right)}^{4}$. $D_{a}$ quantifies the number of dimensions along which the manifold varies significantly. Higher-dimensional manifolds are preferred for minimizing generalization errors. The last term, *signal-noise overlap*, is given by ${\left\|\text{Δ}{\text{x}}_{0}\cdot {\text{U}}_{a}\right\|}^{2}$ and ${\left\|\text{Δ}{\text{x}}_{0}\cdot {\text{U}}_{b}\right\|}^{2}$. Here, ${\text{U}}_{a}\equiv \left[{\text{u}}_{1}^{a}R_{1}^{a},\ldots ,{\text{u}}_{N}^{a}R_{N}^{a}\right]$ and ${\text{U}}_{b}\equiv \left[{\text{u}}_{1}^{b}R_{1}^{b},\ldots ,{\text{u}}_{N}^{b}R_{N}^{b}\right]$ are matrices of the manifold axes of variation. The signal-noise overlap quantifies the alignment between these axes of variation (${\text{U}}_{a}$, ${\text{U}}_{b}$) and the signal direction $\text{Δ}{\text{x}}_{0}$. Smaller overlaps result in lower generalization errors.

We estimate the SNR for each model by first extracting the relational representations of rows associated with each held-out rule, thereby forming the rule manifolds. We then computed the geometric properties of these manifolds and calculated the SNR for each held-out rule pair using Equation 1. Fig. 3B–E presents the computed SNR along with the empirically measured generalization error on the *SimplifiedRPM* task, highlighting close alignments between theoretical predictions (red lines) and empirical results (individual points) across all four models. This alignment validates the geometrical theory as a principled framework for understanding abstract relation generalization through representation geometry.

The geometrical theory identifies three independent mechanisms that facilitate generalization: increasing signal, expanding dimensionality (D), and reducing signal-noise overlap. These mechanisms provide a structured framework for analyzing differences in generalization performance across models. Fig. 3F–I illustrates the geometry of held-out rule representations before (gray bars) and after (colored bars) training, highlighting key differences between models.

Across all models, training increases the SNR for held-out rules (Fig. 3F). When analyzing individual mechanisms, we observe that all models show an increase in signal, with SCL exhibiting a particularly pronounced rise (Fig. 3G). Model-specific differences emerge in dimensionality: ResNet, WReN, and ViT increase D, whereas SCL exhibits a decrease (Fig. 3H). For signal-noise overlap, we normalize the overlap by signal magnitude: $\frac{{\left\|\text{Δ}{\text{x}}_{0}\cdot {\text{U}}_{a}\right\|}^{2}+{\left\|\text{Δ}{\text{x}}_{0}\cdot {\text{U}}_{b}\right\|}^{2}}{{\left\|\text{Δ}{\text{x}}_{0}\right\|}^{2}}$. This normalization allows us to distinguish whether a high signal-noise overlap is due to a large signal magnitude or a genuine alignment between the manifold and the signal direction. We observed that ResNet, WReN, and ViT reduce overlap, whereas SCL shows an increase (Fig. 3I). In summary, ResNet, WReN, and ViT leverage all three mechanisms—enhancing signal strength, expanding D, and reducing overlap—to improve generalization. In contrast, SCL primarily relies on increasing signal strength but does so at the cost of reduced dimensionality and increased overlap.

Previously, we observed that different abstract relations—progression, arithmetic, and logical—exhibited varying levels of generalization error across models (Fig. 2A–D). To understand these differences, we examined the role of signal, dimensionality, and overlap in shaping generalization performance.

**Figure 4:**
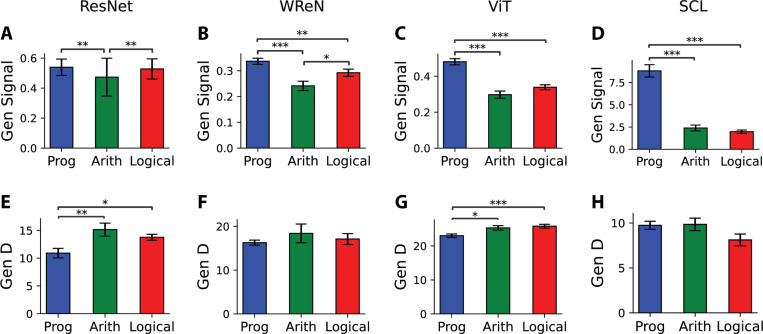
Generalization error differences across abstract relation types were primarily influenced by signal rather than dimensionality. (**A-D**) Generalization signal and (**E-H**) dimensionality for different abstract relation types—progression, arithmetic, and logical—across four models. Results are averaged over 15 random splits of training and held-out rules. Each bar represents the average error for trials where the correct rule of the sample row belonged to each relation type, with error bars indicating the s.e.m. Statistical comparisons were conducted using the pairwise Wilcoxon test.

Across all models, signal strength emerged as the primary driver of generalization performance, with higher signal correlating with lower generalization error. In the SCL model, we observed a human-like generalization pattern where progression rules had significantly lower generalization errors than arithmetic and logical rules (Fig.2D). This was explained by progression rules exhibiting the highest signal (Fig. 4D). A similar pattern was observed in other models, each exhibiting distinct generalization error trends. In ResNet, arithmetic rules’ errors were higher than logical rules due to lower signal (Fig. 4A). In WReN, arithmetic rules had the highest error, driven by the lowest signal (Fig. 4B). In ViT, progression rules had the lowest error, reflecting their highest signal (Fig. 4C).

Beyond signal, dimensionality (D) did not consistently correlate with generalization performance (Fig. 4E–H). In ResNet and ViT, progression rules had the lowest D despite their low errors, suggesting that D alone does not determine generalization ability. Signal-noise overlap also showed no significant difference across abstract relations (figure S3).

### Layer-wise Geometric Analysis Reveals Model-Specific Generalization Strategies

We observed that models exhibit distinct geometries in their relational representations. A key question is how these geometries emerge: Do they form abruptly at specific layers or gradually across the network? To address these questions, we examined the evolution of representational geometry across layers, aiming to pinpoint where abstract relations are extracted and how architecture shapes representation.

**Figure 5:**
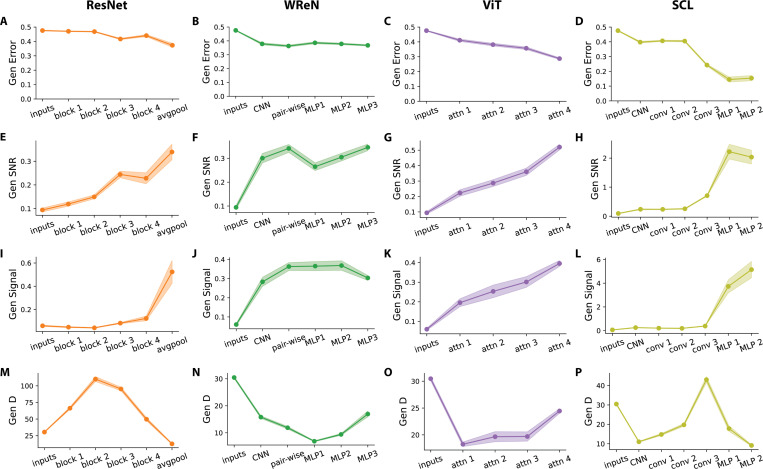
Layer-wise Geometric Analysis Reveals Model-Specific Generalization Strategies. (**A-D**) Generalization error across layers for each model. The plotted values represent 15 distinct held-out rule splits, each containing five held-out rules, leading to 20 rule pairs per split. Error bars denote the s.e.m. across the 15 splits. Representations at each layer are randomly projected to a fixed dimensionality of *N* = 400 before computing errors and performing geometric analysis. Generalization errors for each rule pair are evaluated using 500 *SimplifiedRPM* trials. (**E-H**) Generalization signal-to-noise ratio (SNR) across layers. (**I-L**) Generalization signals across layers. (**M-P**) Generalization dimensionality (D) across layers.

Fig. 5A–D shows each model’s generalization error across layers. At the input stage, rows exhibit a generalization error of 0.475 ± 0.002, close to the 0.5 chance level. This suggests the rules are not yet separated at the row pixel level. Across all four models, generalization errors decrease along the layers, demonstrating progressive refinement of relational representations. Fig. 5E–H shows generalization SNR across layers, highlighting distinct relational development patterns across architectures. ResNet-50 and ViT exhibit a gradual increase in SNR, reflecting a steady accumulation of relational information (Fig. 5E,G). In contrast, WReN plateaus early at the pairwise aggregation layer, with later MLP layers failing to improve further generalization SNR (Fig. 5F). In the SCL model—which achieved the best generalization performance—we observed a sharp increase in generalization SNR at the first MLP layer (Fig. 5H).

To gain deeper insights into how relational rule separability evolves across network layers, we decompose SNR into the dynamics of its geometric terms at each layer. While signal generally increases across layers, its layerwise trend does not always follow that of SNR. While SNR steadily rises in ResNet, the signal remains relatively stable until a sharp increase in the final average pooling layer (Fig. 5I). In WReN, the signal plateaus at the pairwise aggregation layer and even decreases in the final MLP layer (Fig. 5J). In ViT, the signal progressively increases across layers (Fig. 5K). In SCL, the signal only begins to rise sharply in the first MLP layer, whereas SNR increases earlier in the last convolutional layer. Furthermore, while the signal rises in the second MLP layer, SNR unexpectedly decreases (Fig. 5L).

The observed misalignment suggests that signal amplification alone is not sufficient to explain rule separation across layers. To further investigate, we examine the evolution of dimensionality (D) (Fig. 5M–P). Interestingly, unlike the signal, D exhibits a non-monotonic trajectory across layers. ResNet-50 initially expands D in early layers before compressing in later layers (Fig. 5M). WReN follows a different pattern, initially compressing D through pairwise aggregation and early MLP layers before expanding it in later MLP layers (Fig. 5N). ViT maintains a consistently low D throughout, with only a slight increase in the final attention block (Fig. 5O). SCL increases D through convolutional layers but then sharply compresses it in MLP layers (Fig. 5P). These variations in D help explain the gaps observed between signal and SNR evolution through layers. In ResNet-50, the initial expansion of D accounts for the early rise in SNR, which occurs before the signal increases. In SCL, the sharp compression of D in the last MLP layer explains why SNR does not increase alongside the signal.

Across different models, our findings on layerwise geometry suggest that relational abstraction is not a uniform process; each model employs a distinct strategy. The SCL model achieves the best generalization performance by maximizing signal amplification, especially in MLP1, outperforming all other models. In contrast, ResNet-50 and ViT progressively refine relational representations, whereas WReN reaches an early plateau, indicating a bottleneck in relational generalization. While the previous section showed weaker correlations between D and rule separation across abstract relation types, the layerwise analysis reveals a more dynamic interaction. Specifically, D plays a crucial role in shaping relational representations across model layers by refining the effectiveness of signal amplification in driving their emergence.

### Structured Compression Drives the Trade-off Between Generalization Signal and Dimensionality

**Figure 6:**
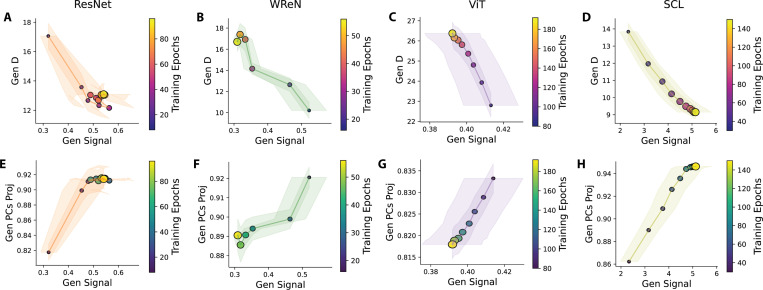
Structured Compression Drives the Trade-off Between Generalization Signal and Dimensionality. (**A-D**) Generalization signal vs. dimensionality (D) across training epochs for all models. Each point shows average signal and D over 15 held-out splits (five rules per split). Dot size and color indicate training epoch; later epochs are larger, brighter. Spearman’s rank correlation between the generalization signal and D reveals a strong negative correlation across the 15 rule splits for each model (ResNet: *ρ* = −0.12±0.12; WReN: *ρ* = −0.41±0.11; ViT: *ρ* = −0.37±0.19; SCL: *ρ* = −0.77 ± 0.05; mean ± s.e.m.). This effect was statistically significant, as confirmed by Stouffer’s test (ResNet: *Z* = 3.41, *p* = 3.2 × 10^−4^; WReN: *Z* = 2.38, *p* = 8.5 × 10^−3^; ViT: *Z* = 14.76, *p* = 1.1 × 10^−49^; SCL: *Z* = 13.04, *p* = 3.5 × 10^−39^). (**E-H**) Alignment of held-out rule manifolds with the low-dimensional subspace of training rules across training epochs for all four models. Each point shows the variance explained by the top 35 principal components of training rule representations for a held-out rule manifold, averaged over 15 splits. The size and color of the dots indicate the training epoch, with later epochs depicted by larger, brighter dots. Spearman’s rank correlation between the generalization signal and subspace alignment shows a strong positive correlation across the 15 rule splits for each model (ResNet: *ρ* = 0.01 ± 0.13; WReN: *ρ* = 0.29 ± 0.11; ViT: *ρ* = 0.38 ± 0.19; SCL: *ρ* = 0.90 ± 0.04; mean ± s.e.m.). This effect was statistically significant in all four models except WReN, as confirmed by Stouffer’s test (ResNet: *Z* = 3.35, *p* = 3.9 × 10^−4^; WReN: *Z* = 0.37, *p* = 0.35; ViT: *Z* = 12.09, *p* = 6.0 × 10^−34^; SCL: *Z* = 19.54, *p* = 2.5 × 10^−85^).We begin plotting from the training epoch, where the structured low-dimensional representation of the training rule first emerges, indicated by the stabilization of its relational representation’s dimensionality. The full trajectory of the generalization signal, D, and alignment is presented in figure S5.

The geometrical theory suggests that high signal and dimensionality (D) benefit generalization. However, we observed that in model layers where significant signal increases occur, D tends to decrease (e.g., Fig. 5I, M for ResNet-50; Fig. 5L, P for SCL). This relationship between the generalization signal and D occurs not only across model layers but also throughout training. In all four models, signal and D exhibit a consistent anti-correlation, with signal increases accompanied by decreases in D (Fig. 6A–D). Intuitively, signal and D are two independent geometric properties. For example, the centroids of two rule manifolds can remain fixed, preserving the signal, while their shapes evolve, changing D. This raises a key question: Why does this trade-off between the generalization signal and D consistently emerge?

We propose that the trade-off between the generalization signal and D arises from a structured compression effect. All class manifolds—including those for held-out rules—gradually align with a low-dimensional subspace defined by the principal components of the training rules. Across models, the representations of the 35 training rules consistently occupy a low-dimensional space. Notably, we observe a drop in variance explained by the top principal components (PCs) at exactly 35, matching the number of training rules (figure S4). We hypothesize that the network amplifies the generalization signal by aligning all representations, including those of held-out classes, within this low-dimensional space.

To test this hypothesis, we measured the fraction of variance explained by the top 35 principal components (PCs) of the training rules for each held-out rule manifold. Higher values indicate more substantial alignment with the low-dimensional subspace of training rules. We observed that as the network signal increases, this alignment becomes more pronounced across models (Fig. 6E–H).

Our findings suggest that the observed signal-D trade-off is not incidental but a consequence of structured compression. As networks enhance the generalization signal, they constrain representations into a low-dimensional subspace, prioritizing inter-class signal at the expense of dimensionality. This effect is a general mechanism across different architectures.

### Theory-Grounded SNR Loss Yields Geometry-Balanced Representations

Can the geometry predicted by the theory directly guide neural network training? We hypothesize that enforcing this geometry provides a principled approach to learning relational representations. To test this hypothesis, we introduce the SNR loss—an SNR-based objective function that explicitly enforces the predicted geometry.

Specifically, the SNR loss maximizes the SNR for the training rule pairs, reinforcing the optimal representation geometry. We sample P rows from m relational rules for each input batch and process them through the model to obtain high-dimensional relational representations. The SNR loss is then formulated as:

(2) $$l_{SNR}=\sum_{\left(a,b\right)}\text{exp}\left(-SNR_{a}\right)$$

Here, $SNR_{a}$ represents the estimated SNR (Equation 1) for the ordered rule pair (a, b), averaged across all possible rule pairs in the batch.

**Figure 7:**
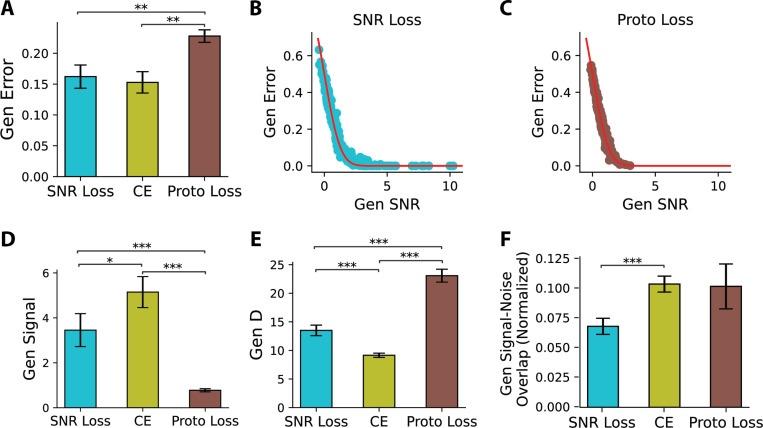
Theory-Grounded SNR Loss Yields Geometry-Balanced Representations. (**A**) Generalization error for models trained with SNR loss, cross-entropy (CE) loss, and Prototypical (Proto) loss, averaged over 15 rule splits. Error bars denote s.e.m. Pairwise Wilcoxon tests confirm Prototypical achieves the highest error (p-values: SNR Loss vs. Proto Loss, 5.1 × 10^−3^; CE vs. Proto Loss, 1.0 × 10^−3^). (**B,C**) Comparison of theoretical predictions (red lines) and empirical generalization errors (dots) for the SNR and Prototypical Loss. Each dot represents a held-out rule pair. (**D-F**) Generalization signal, dimensionality (D), and signal-noise overlap (normalized by signal magnitude) for models trained with SNR, CE, and Proto Loss. Each bar represents the mean value across 15 rule splits, with error bars indicating the s.e.m. Pairwise Wilcoxon tests assess the statistical significance of differences between loss functions. Asterisks indicate statistical significance: * (*p* < 0.05), ** (*p* < 0.01), *** (*p* < 0.001).

To assess the effectiveness of the proposed SNR loss, we trained the top-performing SCL model using this objective and evaluated its generalization across the same 15 rule splits previously used for training with cross-entropy loss. The model effectively optimized the training rule representations, achieving an average SNR of 9.66 ± 0.22 (mean±s.e.m.) across training rule pairs, as evaluated on novel test rows of the training rules. More importantly, the model effectively separated held-out rules, yielding an averaged generalization error of 0.162 ± 0.018 (mean±s.e.m.) (Fig. 7A). This performance was statistically indistinguishable from models trained with cross-entropy (CE) loss using one-hot rule labels (Wilcoxon signed-rank test: statistic = 48.0, *p* = 0.26, n.s.), confirming that SNR loss achieves competitive generalization while structuring learned representations according to theoretical predictions.

To further evaluate the efficacy of SNR loss, we compared its performance against Prototypical loss, a widely used loss function designed to learn structured representations that generalize to unseen classes (26). Prototypical loss operates by embedding inputs into a high-dimensional representation space and forming class prototypes, defined as the centroids of the representations corresponding to each class. The loss function then encourages representations of samples from the same class to cluster around their respective prototypes. Additional details on Prototypical loss are provided in the Supplementary Text. We trained the SCL model using Prototypical loss on the same 15 rule splits. The resulting model exhibited a generalization error of 0.227 ± 0.010, significantly higher than that obtained with SNR loss (Fig. 7A). These findings suggest that while both loss functions promote structured representations, SNR loss is more effective in shaping a geometric representation that enhances generalization.

Notably, the generalization errors of models trained with both SNR loss and Proto loss, similar to our previous observations with cross-entropy loss (Fig. 3E), are also accurately predicted by the geometrical theory (Fig. 7B,C). Compared to cross-entropy (CE) and Prototypical loss, SNR loss is uniquely grounded in geometric theory, explicitly accounting for key representation geometries, including signal strength, dimensionality, and signal-noise overlap. This theoretical foundation raises the question of whether SNR loss yields a more advantageous representational geometry relative to other loss functions. To investigate this, we analyzed the representation geometries induced by different loss functions. Our findings show that representations learned with SNR loss have higher dimensionality than those trained with CE loss (Fig. 7E), addressing the low-dimensionality issue in CE training while maintaining relatively high signal strength (Fig. 7D). Furthermore, SNR loss reduces signal-noise overlap, which is beneficial for generalization (Fig. 7F). Although Prototypical loss also encourages high-dimensional representations, it results in the lowest signal, contributing to its worst generalization performance (Fig. 7D, E). These findings highlight that SNR loss shapes the representations of held-out rules in a way that explicitly adheres to geometric principles.

## Discussion

We address the fundamental question of how neural networks discover unseen rules when learning abstract relational rules. We hypothesize that networks trained to perform reasoning tasks generate good representations even for unseen rules. The utility of these representations can be quantified by the geometry of the *rule manifolds* defined by all representations associated with a given rule. Extending the geometric theory of object manifold (18) to abstract rules, we predicted an explicit relation between the generalization performance in visual reasoning and key geometric properties: manifold radii, dimensionality, the distance between manifold centroids, and the overlap between these distances and their subspaces of variation.

To test whether the geometric theory developed for object recognition can be applied to visual reasoning tasks, we introduce *SimplifiedRPM*, a subset of Raven’s Progressive Matrices (RPM), whose simpler design allows a systematic study of generalization ability in visual reasoning and direct comparison with the above theory. Using this dataset,we trained state-of-the-art deep network architectures (ResNet (16), WReN (2), ViT (17), SCL (5)). We demonstrated the superiority of the SCL (Scatter Compositional Learner) architecture, consistent with previous reports (15). We then conducted an in-depth comparison between the performance of the SCL network and human participants on the same task. Surprisingly, we found excellent agreement between human and network performance, both in terms of overall performance but also across different abstract rules.

Leveraging on a geometrical theory of manifolds initially applied to object recognition tasks (18), we show that interpretable representation geometries can explain models’ performance. Further analysis reveals how different architectures balance signal and dimensionality to achieve relational reasoning while identifying bottlenecks limiting their performance. Building on these insights, we propose SNRloss, a theoretically grounded objective function that explicitly optimizes representation geometry. This novel loss function provides adequate training and generalization capabilities. Our work establishes a geometrically informed framework for analyzing neural networks’ capacity to recognize and generalize abstract relations.

A key insight from our study is that neural networks do not generalize equally well across all abstract relations. While prior research has examined abstract relation generalization in neural networks (2), our work offers a more fine-grained empirical evaluation. Notably, the top-performing model, SCL, aligns closely with human difficulty rankings, with logical rules being the hardest. In contrast, the other three models struggle to generalize arithmetic rules despite achieving high test accuracy. Furthermore, we find that signal, rather than dimensionality or signal-noise overlap, is the primary factor governing these generalization differences among abstract relations. These insights suggest that future models could benefit from strengthening signals for specific relational types.

The best-performing model, SCL, shows a sharp improvement in relational separability only in the last two MLP layers of the network. The earlier layers exhibit only minor gains despite the network being trained end-to-end. This observation suggests that high-level relational abstractions do not gradually build up over the model’s layers but instead emerge abruptly during the final transformations. Geometrically, the signal rises even later in the last MLP layer, while the dimensionality shows a non-monotonic trend—first expanding and then decreasing. The same geometric trend was previously observed in vision models, including ResNet-50, VGG19, and ResNet-152, trained on the ImageNet object classification task (18). The consistency of these geometric trends across models trained on relational reasoning and object classification tasks suggests that they are not merely artifacts of specific architectures or tasks but may reflect a general mechanism of hierarchical abstraction. In contrast, ViTs show a more gradual refinement of relational separation and a steady signal increase across network layers. This indicates that attention mechanisms may support relational learning differently than convolution-based architectures such as SCL and ResNet.

According to the geometrical theory, higher dimensionality should be favorable for generalization. On the other hand, prior studies suggest that low-dimensional manifolds offer advantages for object recognition, enabling the classification of a greater number of objects (27,28). This contrast implies that dimensionality should not be universally maximized or minimized but instead tuned to task demands. Our findings extend this idea by showing that generalization depends not only on dimensionality but mostly on its interaction with pairwise manifold signals. Specifically, we demonstrate that deep networks systematically compress representations into structured, low-dimensional spaces—even for unseen rules. Moreover, this phenomenon is consistently observed across diverse architectures (e.g., ResNet, SCL), indicating that it is a fundamental learning property rather than an artifact of any specific architecture. We observe that late layers play a crucial role in compressing relational representations, a phenomenon consistent with prior work showing that deep networks tend to refine and condense information in later stages of learning (29). These findings open new avenues to explicitly leverage this signal versus dimensionality trade-off to improve generalization.

Among the tested models, SCL best replicates the human-like difficulty ordering across rules. While prior research has relied on explicit relational priors to achieve human alignment (30), our results demonstrate that SCL, when trained end-to-end on the task, exhibits human-like performance without requiring such priors. Furthermore, SCL’s generalization errors closely correlate with human response times. This raises a key question: What insights does SCL offer for understanding the brain’s relational reasoning mechanisms? The SCL network parallels the brain’s hierarchy: its CNN-based feature extractor mimics the ventral visual cortex, capturing basic features like shape and color (31–34), while its later reasoning module reflects higher cortical areas supporting relational reasoning (35–38). We propose that the brain encodes rules of input rows through distinct, rule-specific neural manifolds. Our observation of layer-wise changes in the geometry of these rule manifolds offers hypotheses into how neural representations evolve across brain regions (39,40). According to the SCL model, early visual areas exhibit high representational dimensionality (D) but lowsignal, whereas later regions showlarger signals with reduced dimensionality. These predictions can be empirically tested by measuring neural activity during abstract reasoning tasks (41–44).

This study aimed to study the generalization of abstract relations. While the humans in our experiment were not explicitly trained on the rules, their reasoning abilities could be considered ”pre-trained” through life experiences, as they may have previously encountered similar rules. In contrast, neural networks were trained under strictly controlled rule splits, ensuring they had no prior exposure to the rules evaluated for generalization. To address this discrepancy, a crucial next step is to develop a new benchmark for human reasoning that incorporates abstract relations unlikely to have been encountered. Such a benchmark would disentangle relational generalization from prior experience, providing a clearer view of their distinct roles in human abstract reasoning. This approach enables a direct comparison between humans and neural networks, offering deeper insights into their generalization mechanisms.

In summary, our findings not only advance our understanding of relational reasoning in neural networks but also provide testable hypotheses on how geometric principles may underlie abstract reasoning in the brain. Looking forward, our approach opens exciting opportunities for extending geometric analysis to a broader range of cognitive tasks, including real-world visual reasoning benchmarks and cross-modal linguistic challenges.

## Supplementary Material

Supplement 1

## Data Availability

The code and dataset used in the study are publically available on Github: https://github.com/ShaneShang/SimplifiedRPM-Visual-Reasoning.
