# Supplementary material for: Unraveling the Geometry of Visual Relational Reasoning
Source: ArXiv. 2025 Jul 25:arXiv:2502.17382v2. Originally published 2025 Feb 24. Preprint. [Version 2] (PMC11888560)
Supplement: Supplement 1 [file NIHPP2502.17382v2-supplement-1.pdf]

Supplementary Information

Unraveling the Geometry of Visual Relational Reasoning

Jiaqi Shang, Gabriel Kreiman, and Haim Sompolinsky

**Correspondence: hsompolinsky@mcb.harvard.edu*

A Supplementary Methods

A.1 Model Architecture and Hyperparameter Tuning

We used the standard ResNet-50 architecture described by[28] without any modifications. The architecture comprises residual blocks containing convolutional layers followed by batch normalization and ReLU activation. Specifically, the model begins with an initial convolutional layer, followed by four residual blocks, and concludes with a global average pooling layer and a fully connected layer for classification. To analyze the evolution of layer-wise geometry, we extracted features from the outputs of each residual block (Block 1–4) and the final output of the average pooling layer (avgpool).

The Wild Relation Network (WReN) model captures and integrates pairwise relational information from the three panels in an input row. Each panel is first processed through a shared four-layer convolutional block to generate an individual panel embedding. Then, embeddings from each pair of panels are concatenated and passed through a multi-layer perceptron (MLP). The resulting pairwise features are summed to form an aggregated relational representation, which is further refined through three additional MLP layers. For layer-wise geometric analysis, we extract representations from the convolutional block output (CNN), the aggregated pairwise features (Pairwise), and the three successive MLP layers (MLP1–3).

For hyperparameter tuning of WReN, we performed a grid search over the following parameters: (1) hidden dimension of the pairwise relational module $\{128, 256, 512\}$; (2) number of layers in the pairwise relational module $\{1,2,3,4\}$; (3) hidden dimension of the classification MLP $\{64, 128, 256\}$; and (4) number of layers in the classification MLP $\{1,2,3,4\}$. The optimal configuration, minimizing test error on three held-out rule splits, was found to be a pairwise relational module with 2 layers of 512 dimensions and a classification MLP with 3 layers of 256 dimensions.

The Vision Transformer (ViT) architecture comprises multiple layers of multi-headed self-attention (MSA). In our implementation, we utilize a four-layer Transformer model, each containing 12 attention heads and a hidden dimension of 96. The model processes input row patches, embedding them into token representations with a learnable class token prepended to the sequence. These representations are then sequentially processed through self-attention layers to capture global contextual dependencies. For layer-wise geometric analysis, we extract the outputs from each attention layer (Attn1-4).

For hyperparameter tuning of ViT, we performed a grid search over the following parameters: (1) number of layers $\{4, 8, 12\}$; (2) number of attention heads $\{4, 8, 12, 16\}$; (3) hidden dimension of the self-attention layers $\{48, 96, 192, 384, 768\}$. The optimal configuration, determined by minimizing test error, was found to be 4 layers, each with 12 heads and a hidden dimension of 96.

The Scattering Compositional Learner (SCL) model[9] is a hierarchical architecture designed explicitly for RPM tasks (figure S6). Each input panel is first processed through a shared convolutional block, producing an individual panel embedding. These embeddings are then processed through three 1-D convolutional layers that operate across the three panels. Crucially, the same filter is applied consistently across the panel-wise feature dimensions, ensuring that abstract relations are detected in an attribute-invariant manner. Finally, the output is further refined using two MLP layers. For layer-wise geometric analysis, we extract representations from the convolutional block (CNN), the three 1-D convolutional layers (conv1-3), and the two MLP layers (MLP1–2).

For hyperparameter tuning of SCL, we performed a grid search over the following parameters: (1) the kernel size of the 1-D convolutional layers $\{1,2,4,8\}$; (2) the number of output channels, chosen from $\{16, 32\}$ for the first layer, $\{32, 64\}$ for the second layer, and $\{5, 10\}$ for the final layer; (3) the hidden dimensions of the last two layers of the multi-layer perceptron (MLP) $\{400, 1000\}$. The optimal configuration, determined by minimizing test error, was found to be a kernel size of 1, output channel sizes of $\{64, 32, 5\}$, and an MLP hidden dimension of 400.

A.2 Prototypical loss and SNR loss

For the SNR loss, we conduct a grid search over batch composition and learning rate. Following the setup used in cross-entropy training, we maintain a total batch size of 512 and explore three batch compositions: $\{32\times 16, 16\times 32, 8\times 64\}$ (m classes $\times$ P samples per class). Additionally, we test three learning rates: $\{0.1, 0.01, 0.001\}$. Model performance is evaluated based on

test error across three held-out rule splits, distinct from the 15 used for training. The optimal configuration is 32×16 with a learning rate of 0.01.

The prototypical loss[33] is a distance-based loss function commonly used in few-shot learning. It generates rule prototypes, which act as representative representations for each rule. Like the SNR loss, each input batch consists of P rows drawn from m different relational rules. For each rule r, the P rows are randomly split into two disjoint subsets: a support set S_r and a query set Q_r. The prototype z_r for rule r is computed as the mean representation of its support set:

$$z_r = \frac{1}{| S_r |} \sum_{x \in S_r} x \tag{3}$$

where x denotes the relational representation of an input row.

The prototypical loss encourages rows in the query set Q_r to be closer to their corresponding rule prototype z_r while pushing them away from prototypes of other rules $z_{c'} (c' \neq c)$ in the batch. It is formulated as:

$$l_{\text{Prototypical}} = \sum_{r} \sum_{x \in Q_c} -\log \frac{\exp\left(- \| x - z_c \|^2\right)}{\sum_{c' \neq c} \exp\left(- \| x - z_{c'} \|^2\right)} \tag{4}$$

To optimize the Prototypical loss, we conducted a grid search over: (1) batch size $\{32 \times 16, 16 \times 32, 8 \times 64\}$ (m classes $\times$ P samples per class); (2) learning rate $\{0.1, 0.01, 0.001\}$, and support set size $\{1, 5\}$. Model performance was assessed using test accuracy on three held-out rule splits, distinct from the 15 used for training. The optimal configuration was found to be 32 classes $\times$ 16 samples per class, with a learning rate of 0.01 and support set size 5.

B Supplementary Figures

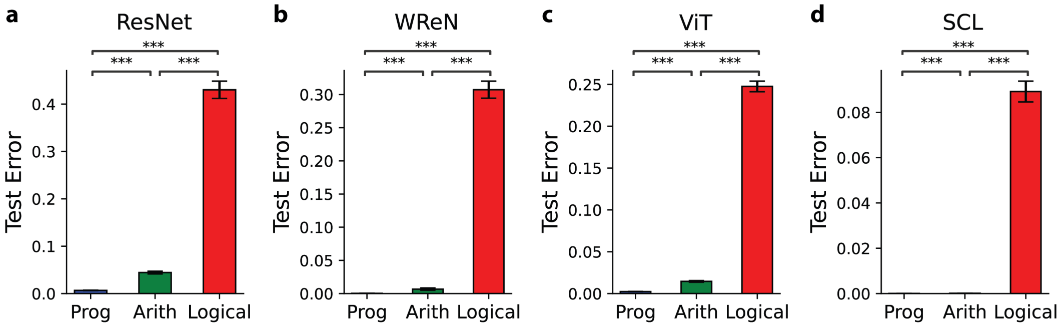

Figure S1. Model Test Errors Across Abstract Relation Types. (a-d) Test errors of four models on the 35 training rules, evaluated across three abstract relation types: Progression (blue), Arithmetic (green), and Logical (red). The chance error is 0.97. Each bar shows the mean classification error for rows of rules belonging to one of these relation types. Error bars represent the standard error of the mean (s.e.m.). Statistical significance was assessed using pairwise Wilcoxon tests, with asterisks indicating significance levels: *** ($p < 0.001$).

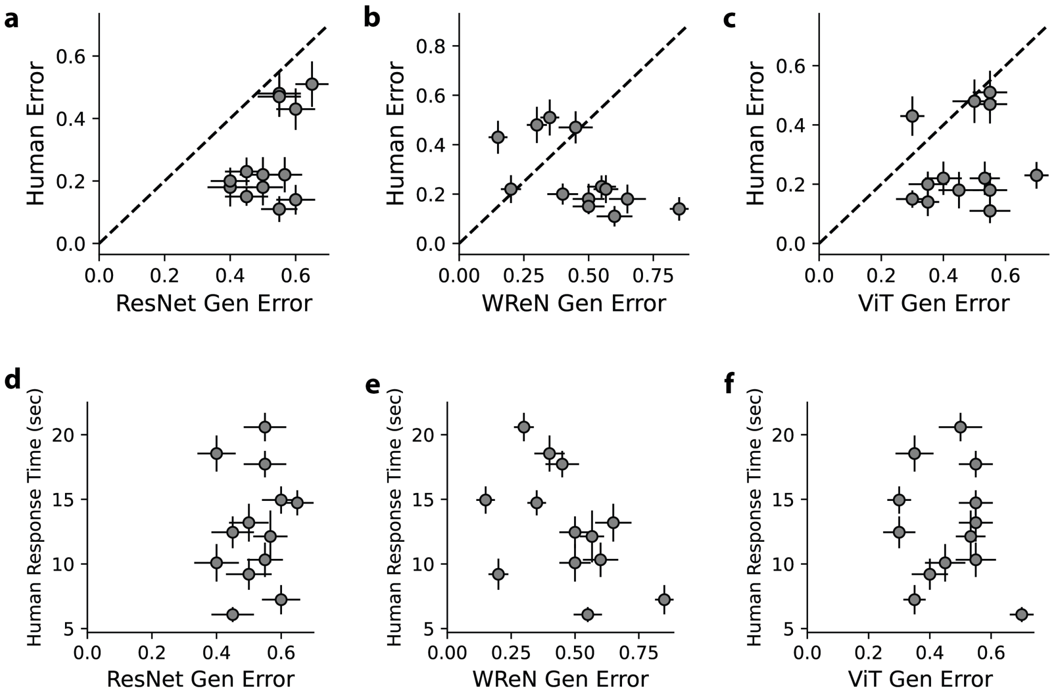

Figure S2. Comparison of Model Generalization Errors with Human Performance on the *SimplifiedRPM* Task. (a-c) Comparison of the generalization errors of ResNet-50, WReN, and ViT models with human performance on the *SimplifiedRPM* task across 13 selected rule pairs. Each dot represents the average model error (x-axis) and human error (y-axis) over 10 questions for a given rule pair, with error bars indicating the standard error of the mean (s.e.m.). The dashed line represents the line of identity. No significant positive correlation is observed between model and human errors (Spearman's rank correlation: ResNet-50, $\rho = 0.34$, $p = 0.24$; WReN, $\rho = -0.67$, $p = 0.01$; ViT, $\rho = 0.27$, $p = 0.37$). (d-f) Relationship between model generalization error (x-axis) and human response time (y-axis) for the 13 selected rule pairs in ResNet-50, WReN, and ViT models. Each dot represents the average model error and corresponding human response time for a given rule pair, with error bars indicating the s.e.m. No significant correlation is found between model error and human response time (Spearman's rank correlation: ResNet-50, $\rho = 0.12$, $p = 0.68$; WReN, $\rho = -0.50$, $p = 0.08$; ViT, $\rho = -0.14$, $p = 0.65$).

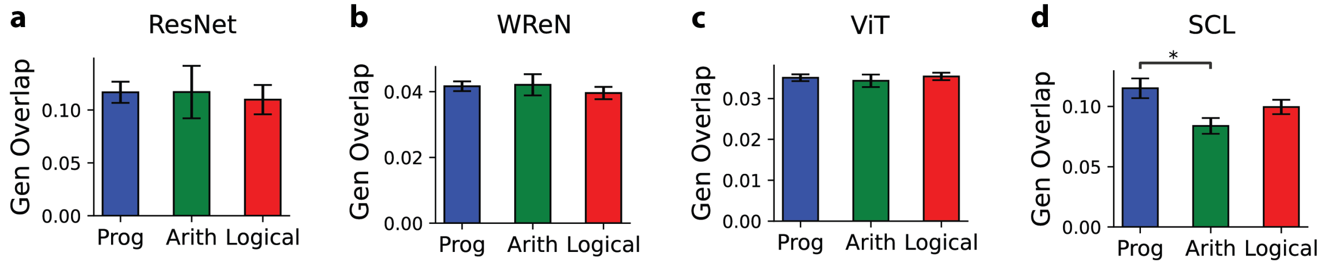

Figure S3. Generalization signal-noise overlap differences across abstract relation types. (a-d) Generalization signal-noise overlap (normalized by signal magnitude) for different abstract relation types—progression, arithmetic, and logical—across four models. Results are averaged over 15 random splits of training and held-out rules. Each bar represents the average error for trials where the correct rule of the sample row belonged to each relation type, with error bars indicating the s.e.m. Statistical comparisons were conducted using the pairwise Wilcoxon test. Asterisks indicate statistical significance: * ($p < 0.01$).

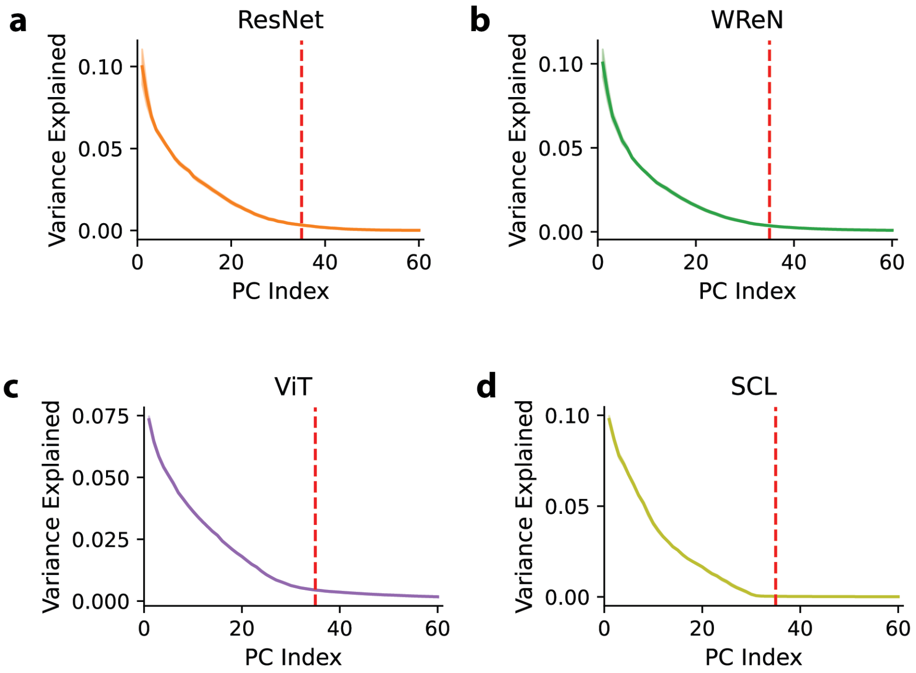

Figure S4. Fraction of variance explained by the principal components (PCs) of the training rule representations. (a-d) We aggregate the relational representations of the 1,000 test rows for each of the 35 training rules and perform principal component analysis (PCA) on these representations. For each of the four neural networks under study, we plot the fraction of variance explained by each principal component (PC), displaying only the top 60 PCs. The red line indicates the 35th PC, corresponding to the number of training rules. A noticeable drop in explained variance at this point suggests that the representations of the 35 training rules are consistently confined to a low-dimensional subspace. The lines represent the average explained variance across 15 random splits of held-out rules, with shading indicating the standard error of the mean (s.e.m.).

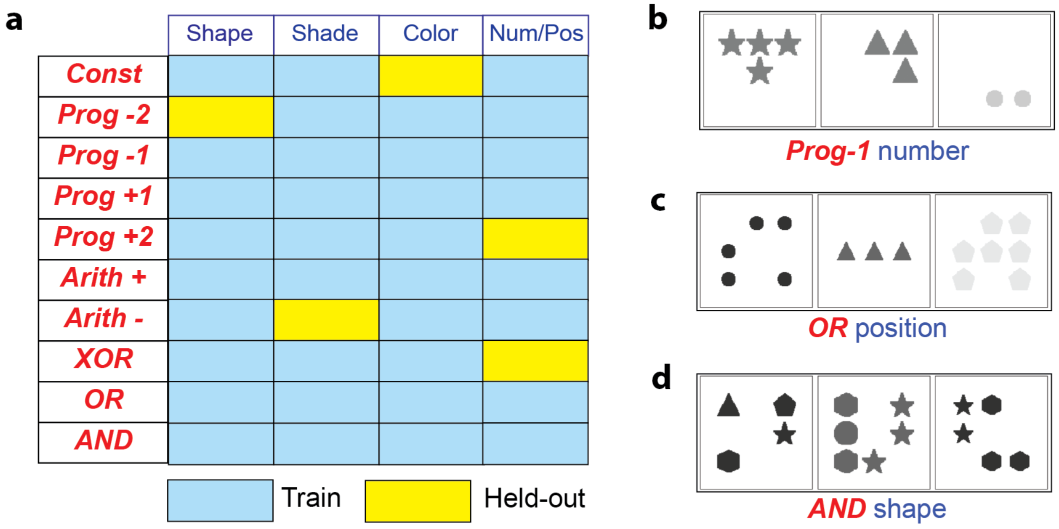

Figure S5. Overview of *SimplifiedRPM* dataset rules and example rows. (a) Rules in the *SimplifiedRPM* dataset. Each rule in the *SimplifiedRPM* dataset is defined by an abstract relation (first column) applied to a specific object attribute, resulting in a total of 40 possible rules. During training, we create rule splits by randomly selecting five rules to be held out, ensuring that each abstract relation appears at most once in the held-out set. The table illustrates an example of this split, where yellow cells indicate the five held-out rules, and blue cells represent the remaining 35 rules used for training the models. (b-d) Example rows in the *SimplifiedRPM* dataset. (b) Example row illustrating the Progression -1 number relation, where the number of objects in each panel decreases by one across the row (4 objects in the first panel, 3 in the second, and 2 in the last). (c) Example row demonstrating the OR position rule, where objects in the third panel occupy positions in either the first or second panel. (d) Example row illustrating the AND shape rule, where the third panel contains shapes (hexagon, star) that appear in both the first and the second panel.

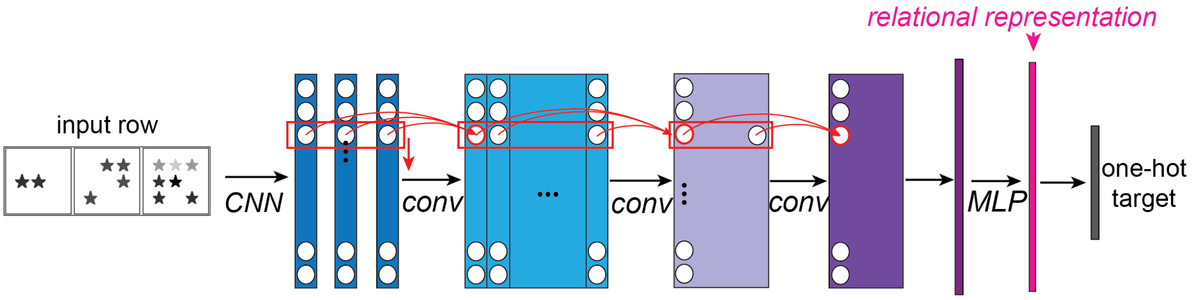

Figure S6. Overview of the SCL architecture. Each input row, consisting of three panels, is processed through three 1-D convolutional layers, followed by two fully connected multilayer perceptron (MLP) layers. The final output is of size 35, compared against the one-hot target vector corresponding to the 35 possible relational rules. The penultimate layer is the relational representation used for the *SimplifiedRPM* task.

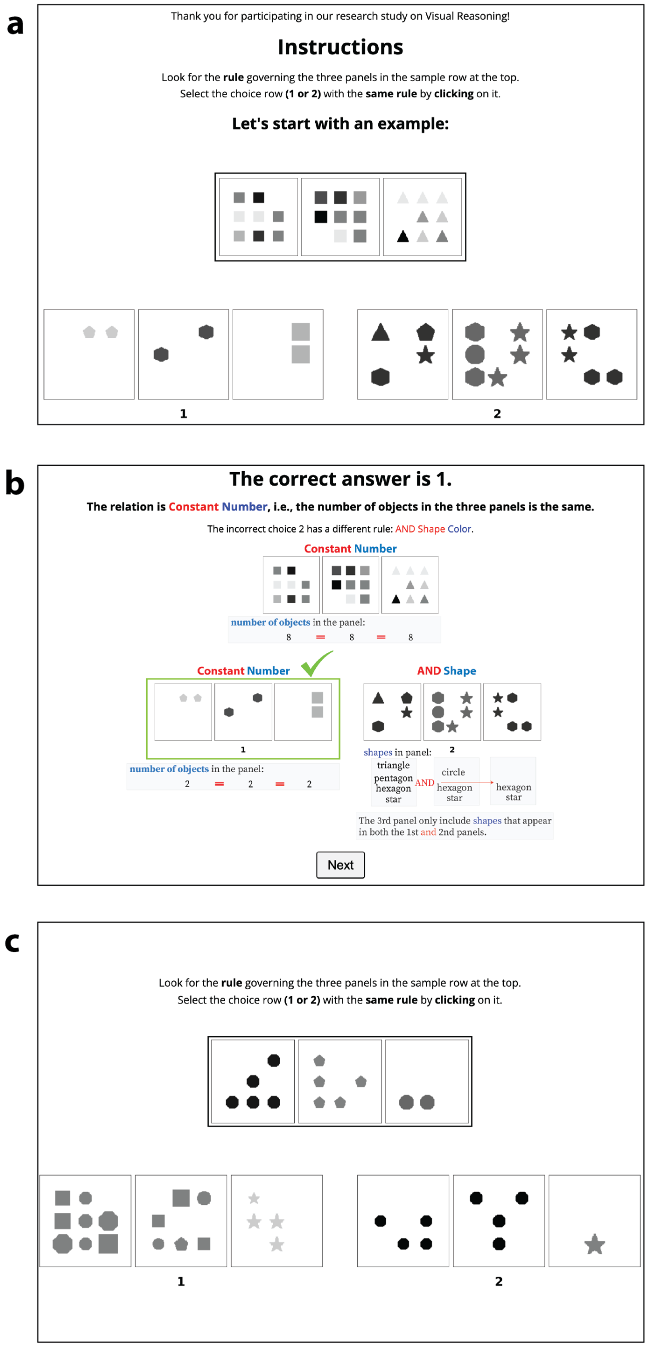

Figure S7. Example trials in the human experiment. (a) Instruction and practice trial, where participants are introduced to the task. (b) Explanations are provided to participants after they make a selection in the practice trial. (c) Example trial from the main experiment, where no feedback is given.

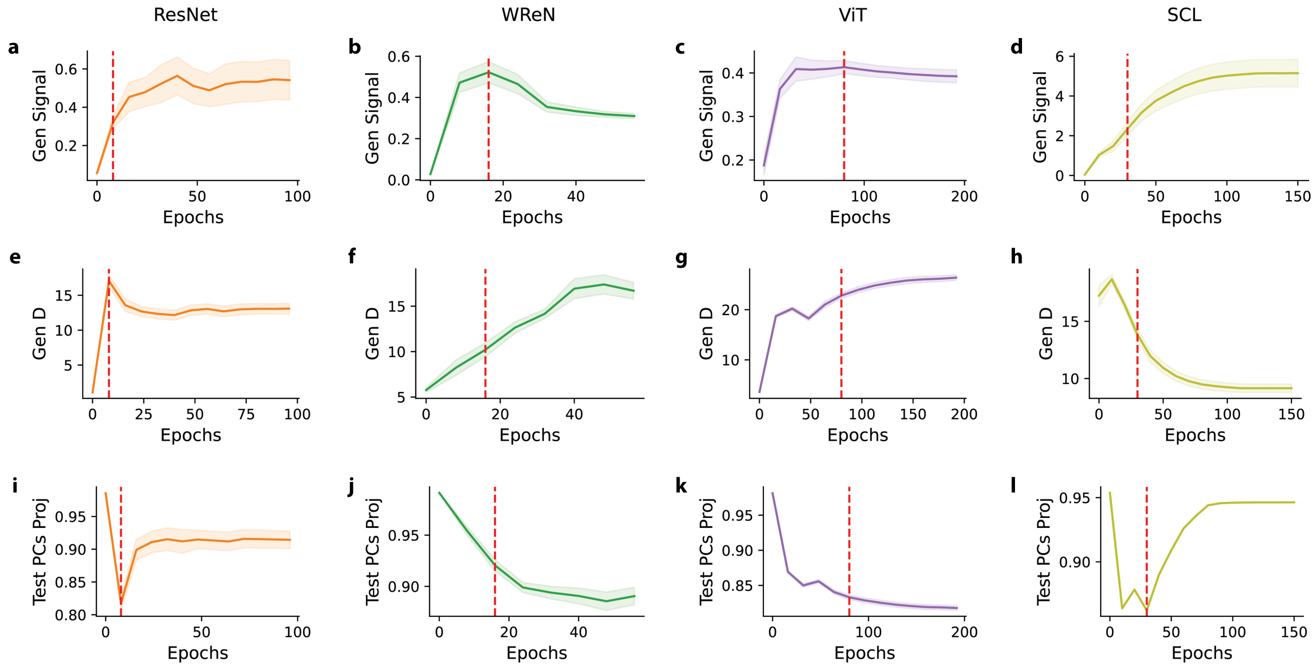

Figure S8. Comparison of Model Generalization Errors with Human Performance on the *SimplifiedRPM* Task. Changes in generalization signal (a-d), generalization dimensionality D (e-h), and the variance of held-out rule manifolds explained by the top 35 principal components of training rules (i-l) over training epochs. Results are averaged across 15 different held-out rule splits, with shaded areas representing the standard error of the mean (s.e.m.) for the four models (ResNet, WReN, ViT, and SCL). The red vertical line marks the epoch at which the relational representation structure for the training rules stabilizes.

C Supplementary Tables

Table S1. Human Performance on 13 Rule Pairs. Thirteen rule pairs were tested in the human experiment. The correct rule corresponds to the rule governing both the sample row and the correct choice row, while the incorrect rule applies to the incorrect choice row. Human error represents the average error rate across 10 trials for each rule pair, with error bars indicating the standard error of the mean (s.e.m.). We recruited 25 MTurk participants (n=25), each completing a subset of 60 trials from a total of 130 (13 rule pairs $\times$ 10 trials per rule pair). The error for each trial was calculated as the mean response error across 10 participants. Response time denotes the total duration from trial onset (when the stimulus appears on the screen) to the moment the participant selects one of the two choice images. Rule pairs are ordered by average human error.

#	Correct Rule	Incorrect Rule	Human Error	Response Time (sec)
1	Prog-1 Number	AND Shape	0.11 ± 0.04	10.32 ± 1.33
2	Prog+1 Number	Const Number	0.14 ± 0.05	7.23 ± 1.13
3	OR Shape	Arith+ Number	0.15 ± 0.03	13.52 ± 1.08
4	Prog+2 Number	Const Number	0.18 ± 0.06	10.08 ± 1.45
5	Arith- Number	OR Shape	0.18 ± 0.06	13.19 ± 1.46
6	XOR Shape	AND Position	0.20 ± 0.04	18.57 ± 1.57
7	Prog-2 Number	XOR Position	0.22 ± 0.06	8.87 ± 1.23
8	Arith+ Number	OR Position	0.22 ± 0.06	12.13 ± 2.01
9	Const Number	Prog+2 Number	0.23 ± 0.04	6.08 ± 0.58
10	AND Position	Prog-2 Number	0.43 ± 0.07	14.95 ± 1.05
11	AND Shape	XOR Shape	0.47 ± 0.06	17.80 ± 1.07
12	OR Position	XOR Position	0.48 ± 0.07	20.62 ± 1.18
13	XOR Position	Arith- Number	0.51 ± 0.07	14.71 ± 1.02

Table S2. Relations in the *SimplifiedRPM* Dataset. The *SimplifiedRPM* dataset includes ten abstract relations organized into progression, arithmetic, and logical types. Each rule defines how attribute index values assigned to objects change across three panels. Each example consists of three lists, each representing a panel and specifying the assigned attribute values. For instance, $\{[1],[1],[1]\}$ represents a constant relation, with objects maintaining the attribute value one across all panels. Objects can also have multiple attribute values, as seen in the *AND* relation example $\{[1],[0,1],[1]\}$, where the second panel contains objects with attributes 0 and 1.

Relation Type	Relation	Description	Example
**Progression**	Const	Attribute values are the same across all three panels.	{[1], [1], [1]}
	Prog -2	Attribute values decrease incrementally by 2 across the panels.	{[4], [2], [0]}
	Prog -1	Attribute values decrease incrementally by 1 across the panels.	{[2], [1], [0]}
	Prog +1	Attribute values increase incrementally by 1 across the panels.	{[0], [1], [2]}
	Prog +2	Attribute values increase incrementally by 2 across the panels.	{[0], [2], [4]}
**Arithmetic**	+	Attribute values in the 3rd panel are the sum of the values in the first two panels.	{[0], [1], [1]}
	-	Attribute values in the 3rd panel are the first panel's values minus the second panel's values.	{[1], [1], [0]}
**Logical**	AND	3rd panel contains attribute values present in both the first and second panels.	{[0], [0, 1], [0]}
	OR	3rd panel contains attribute values present in either the first or second panel.	{[0], [0, 1], [0, 1]}
	XOR	3rd panel contains attribute values present in only one of the first two panels.	{[0], [0, 1], [1]}
